# Serum D‐2‐hydroxyglutarate and the ratio of D‐2HG/L‐2HG predict *IDH* mutation in acute myeloid leukemia

**DOI:** 10.1002/jha2.723

**Published:** 2023-06-19

**Authors:** Chelsea Zhang, Andrew Chu, Richard Boriack, Robert Collins, Jing Xu, Dwight Oliver, Ruifang Zheng, Dinesh Rakheja, Weina Chen

**Affiliations:** ^1^ Departments of Pathology University of Texas Southwestern Medical Center Dallas Texas USA; ^2^ Children's Medical Center Dallas Dallas Texas USA; ^3^ Internal Medicine (Hematology and Oncology) University of Texas Southwestern Medical Center Dallas Texas USA

**Keywords:** acute myeloid leukemia, D‐2HG/L‐2HG Ratio, D‐2‐Hydroxyglutarate, IDH, L‐2‐Hydroxyglutarate

## Abstract

This study investigates whether serum D‐2HG (D‐2‐hydroxyglutarate) produced by the mutated isocitrate dehydrogenase (IDH) can predict *IDH* mutations in acute myeloid leukemia (AML) at diagnosis. D‐2HG and L‐2HG are measured by liquid chromatography‐tandem mass spectrometry. D‐2HG, total 2HG and the D/L ratio (D‐2HG/L‐2HG) are significantly higher in *IDH* ^mutated^ cases than in *IDH* ^wild^ cases. The optimal cutoff values to predict *IDH* mutations at 100% sensitivity (specificity 91%–94%) are >588 ng/mL for D‐2HG and >2.33 for the D/L ratio. Our study indicates that elevated serum D‐2HG and the D/L ratio may serve as noninvasive biomarkers of *IDH* mutation in AML.

## 1 INTRODUCTION

Mutations in genes encoding isocitrate dehydrogenase (IDH1 and IDH2) in acute myeloid leukemia (AML) result in loss‐of‐function for the oxidative decarboxylation of isocitrate to α‐ketoglutarate (α‐KG) and confer a novel catalytic activity that reduces α‐KG to the D‐enantiomer of 2‐hydroxyglutarate (D‐2HG) [1, 2, 3, 4, 5, 6, 7]. All known *IDH* mutations involve arginine (R), in the codon 132 of *IDH1* or codon 140 or 172 of *IDH2*. The oncogenic property of D‐2HG appears to stem from its ability to inhibit α‐KG‐dependent dioxygenases, including histone demethylases, Ten‐eleven translocation hydroxylases and prolyl hydroxylases, thus initiating dysregulated epigenetic programming and hypoxia inducible transcription factor (HIF)‐1α pathway. The determination of *IDH* mutation status in AML has clinical relevance in treating AML patients harboring the *IDH* mutations by the IDH inhibitor and monitoring response to conventional as well as IDH‐targeted therapy.

D‐2‐hydroxyglutarate (D‐2HG) and L‐2‐hydroxyglutarate (L‐2HG) are two enantiomers of 2‐hydroxyglutarate (2HG). They are normal endogenous metabolites that can be oxidized back to α‐KG by their respective 2‐hydroxyglutarate dehydrogenases (D‐2HG dehydrogenase and L‐2HG dehydrogenase, respectively). As such, D‐2HG and L‐2HG levels are at homeostasis but could be affected by abnormal enzymatic activities with associated alterations in these genes. Notably, *IDH* mutations only produce D‐2HG. Several studies have found that elevated 2HG in plasma or serum (either total 2HG [D‐2‐HG + L‐2HG] or without specifying enantiomer type) could serve as a biomarker of *IDH* mutations [8, 9, 10, 11, 12]. However, only rare studies reported the ratio of D‐2HG/L‐2HG (referred to as the D/L ratio) and reporting on D‐2HG is largely absent.

Our study aims to systematically investigate whether increased D‐2HG, total 2HG, and the D/L ratio can predict *IDH* mutations in AML at diagnosis. Our results indicate at the optimal cut‐offs with 100% sensitivity, D‐2HG, total 2HG, the D/L ratio can predict *IDH* mutations with a high specificity (91%–94%). Additionally, we report unusual and novel findings, two cases with increased D‐2HG but lacking *IDH* mutation and the first case of elevated L‐2HG in AML. The significance of these findings will be discussed.

## 2 METHODS

Peripheral blood specimens (53 cases collected in 2015) were obtained from newly diagnosed or refractory AML patients at the Hospitals of The University of Texas Southwestern Medical Center. None of these patients were treated with an IDH inhibitor. This study was approved by the institutional review board. *IDH* mutation status was assessed by polymerase chain reaction amplification of segments of *IDH* exon 4 and flanking intronic regions followed by Sequenom mass spectrometry (targeting *IDH1* R132 and *IDH2* R140/172, with a detection sensitivity of ∼10% variant allelic frequency [VAF]), and by FoundationOne next‐generation sequencing (NGS) panel (with a detection sensitivity of ∼3% VAF) in a small subset of cases. The variant of allelic frequency in *IDH* mutation was not available in most cases.

The levels of two enantiomers, D‐2HG and L‐2HG, were measured by liquid chromatography‐tandem mass spectrometry [13]. All data were analyzed by Prism (Version 9). A *p* value less than 0.05 was considered significant. Of 20 patients with refractory AML, most patients underwent standard chemotherapy (7 + 3). While the levels of D‐2HG are expected to be affected due to blast count changes following chemotherapy, the key results and conclusion are not expected to be affected because the inclusion criteria were AML with the blast counts generally at the leukemia range, and there is no report of standard chemotherapy agents specifically targeting *IDH* genes.

## 3 RESULTS

### 3.1 Clinicopathological features of AML patients with and without *IDH* mutations

Clinical characteristics of 53 AML patients (33 males and 20 females, and aged 31–85 years) with and without *IDH* mutations are summarized in Table 1. There was no difference in age, gender, white cell count, or blast% in peripheral blood (PB), the frequencies of abnormal karyotype and complex karyotype between the two groups. *IDH* mutations were not associated with any particular karyotypic abnormality while *IDH* unmutated cases had recurrent cytogenetic abnormalities, t(15;17)(q24;q21.1), inv(16)(p13q22), and t(6;9)(p23;q34), each in one case. Nineteen AML cases (36%) were *IDH* mutated (*IDH^m^*), most as *IDH1* R132 (cases with R132C/G/S) and *IDH2* R140Q (eight cases; only three cases with *IDH2* R172K), while 34 cases (64%) were *IDH* wild‐type (*IDH^w^*).

**TABLE 1:** Clinical characteristics of AML patients with and without *IDH* mutations.

	mutated *IDH* (*n* = 19)	wild‐type *IDH* (*n* = 34)	*p‐*Values
Age, year	65 (53–81)	72 (31–85)	0.9755
Male/female ratio	13 M/6F	22 M/14F	0.7695
PB blast %	8.0% (1.0%–95%)	10% (2.0%–9.5%)	0.8985
WBC (×10^9^/L)	3.3 (0.5–181)	2.7 (0.2–125)	0.9812
Abnormal CG	47%	53%	0.7786
Complex karyotype	26%	15%	0.4653

*Note*: All values except the M/F ratio are median and range.

Abbreviations: CG, cytogenetics; F, female; M, male.

### 3.2 High D‐2HG, total 2HG, and the D/L ratio to predict *IDH* mutations (establishment of cut‐off values)

**FIGURE 1:**
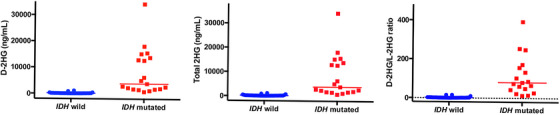
Increased serum D‐2HG, total 2HG and the D/L ratio *in IDH* mutated AML. D‐2HG, total 2‐HG and the D/L ratio are significantly higher in *IDH^m^* cases [median (indicated by bar) of 3,815 ng/mL, 3,852 ng/mL and 80.6, respectively)] than in *IDH^w^* cases (67 ng/mL, 138 ng/mL and 1.27, respectively).

D‐2HG, total 2HG, and the D/L ratio were significantly higher in *IDH^m^* cases (median of 3815 ng/mL, 3852 ng/mL, and 80.6, respectively) than in *IDH^w^* cases (67 ng/mL, 138 ng/mL, and 1.27, respectively, *p* < 0.001; Table 2, Figure 1). There was no difference in these values among the *IDH* mutation types. The L‐2HG levels were not different between the two groups.

**TABLE 2:** D‐2HG, L‐2HG and the D/L ratio in AML patients with and without *IDH* mutations and the cut‐offs to predict *IDH* mutations.

Median (range)	D‐2HG (ng/mL)	Total 2HG (ng/mL)	2HG D/L ratio	L‐2HG (ng/mL)
Reference ranges in PB	18–263	60–375	0.222–3.77	6–147
AML, *IDH ^w^* (*n* = 34)	67 (24–1,120)	138 (47–1,192)	1.27 (0.2–15.6)	58 (20–651)
AML, *IDH ^m^* (*n* = 19)	3,815 (734–34,200)*	3,852 (761–34,266)*	80.6 (13.2–518)*	61 (26–147)
Cut‐off values for 100% sensitivity	>588	>623	>2.33	
Specificity (CI) at 100% sensitivity	94% (81%–99%)	92% (78%–98%)	91% (82%–100%)	

*Note*: 2HG unit: ng/mL (molecular weight 148.1 g/mol).

Abbreviation: CI, confidence interval.

* *p* < 0.0001 comparing the corresponding values in mutated versus wild type *IDH*.

**FIGURE 2:**
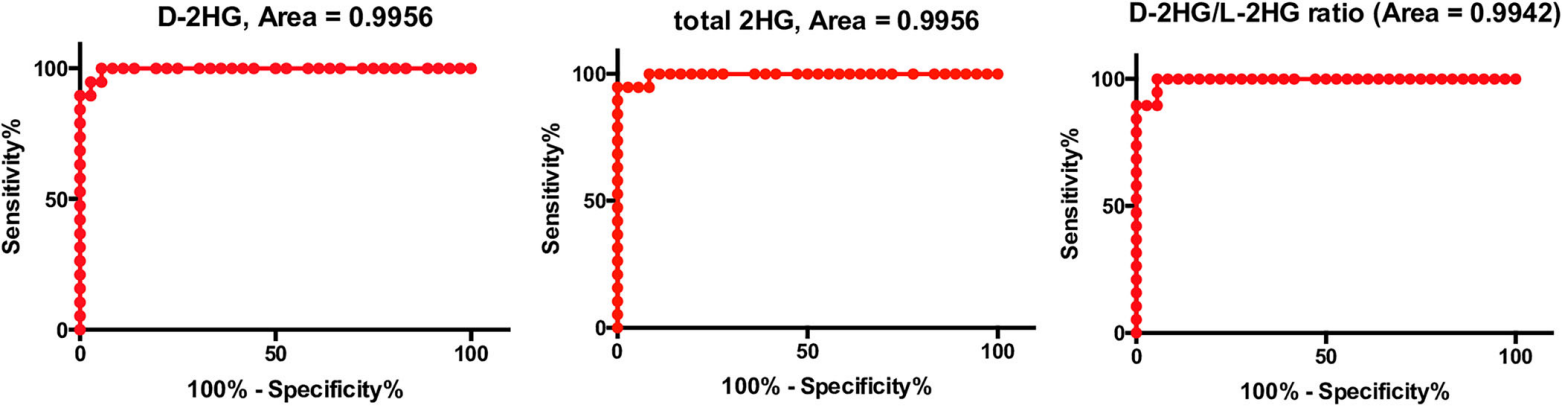
Diagnostic performances of D‐2HG, total 2HG and the D/L ratio to predict *IDH* mutations indicated by the ROC curves. The optimal diagnostic cut‐off values for D‐2HG, total 2HG and the D/L ratio that ensure maximum specificity at 100% sensitivity to predict *IDH* mutation are determined by area under the receiver operating characteristic curve (ROC AUC). The cut‐off values are >588 ng/mL for D‐2HG (with a specificity of 94%, AUC 0.996), >623 ng/mL for total 2HG (with a specificity of 92%, AUC 0.996) and >2.33 for the D/L ratio (with a specificity of 91%, AUC 0.994).

The optimal diagnostic cut‐off values for D‐2HG, total 2HG, or the D/L ratio that ensure maximum specificity at 100% sensitivity to predict *IDH* mutation were determined by area under the receiver operating characteristic curve (ROC AUC). The cut‐off values were > 588 ng/mL for D‐2HG (with a specificity of 94% in this dataset, AUC 0.996), >623 ng/mL for total 2HG (with a specificity of 92%, AUC 0.996), and >2.33 for the D/L ratio (with a specificity of 91% in this dataset, AUC 0.994) (Table 2 and Figure 2). These results indicated that elevated serum D‐2HG, total 2HG, and the D/L ratio could be used as highly sensitive and specific biomarkers (91‐94% specificity at 100% sensitivity) of *IDH* mutation in AML.

### 3.3 Unusual cases with *discordancy* between *IDH* mutation and the D/L ratio/2HG levels

Several notable findings in this study merit further description (Table 3). Firstly, case 1 with *IDH2* R140Q mutation (3% VAF and 4% blasts in PB, not included in the cut‐off established series) had only elevated D/L ratio (10.0) but the D‐2HG (441 ng/mL) and total 2HG (485 ng/mL) levels below the diagnostic cut‐offs. Secondly, cases 2 and 3 without *IDH* mutation (by NGS panel and ∼30% blasts in PB) had elevated D/L ratio (∼15) and D‐2HG level (∼1000 ng/mL) much higher than the diagnostic cut‐offs. Lastly, case 4 without *IDH* mutation (by NGS panel) had an unexpectedly high‐level of L‐2HG (651 ng/mL). Understanding these apparently *discordant* results between *IDH* mutation status and the D/L ratio and 2HG levels may provide insights into the significance of these biomarkers.

**TABLE 3:** Unusual cases of AML with “discordancy” between *IDH* mutation status and 2HG and the D/L ratio levels.

Cases	Blast% in PB	*IDH* Mutation (by NGS)	D‐2HG (ng/mL)	Total 2HG (ng/mL)	D/L ratio	L‐2HG (ng/mL)
1	4%	Positive (*IDH2* R140Q, VAF 3%)	**441**	485	**10.0**	44
2	22%	Negative	**1120**	1,192	15.3	72
3	41%	Negative	**887**	945	15.6	58
4	8%	Negative	130	781	0.20	**651**

Abbreviation: VAF, variant allelic frequency.

## 4 DISCUSSION

Our study demonstrates that elevated serum D‐2HG (>588 ng/mL), total 2HG (>623 ng/mL), and the D/L ratio (>2.33) are highly sensitive and specific biomarkers (91%–94% specificity at 100% sensitivity) of *IDH* mutation in AML at diagnosis. These cut‐off values are generally comparable to the levels established in other studies, such as the D/L ratio of 1.9–2.5 in two European studies [8, 9] and 2HG level of ∼500–600 ng/mL (without specifying enantiomer type) in two US studies [10, 12], but a much lower 2HG level of ∼300 ng/mL in one European study [8]. Additionally, our study seems to be the first to report the D‐2HG threshold. Comparing the D/L ratio of 2.33 from our study to those reported, a cut‐off of 1.9 will maintain a 100% sensitivity but reduce the specificity from 91% to 79% while a cut‐off of 2.5 will result in a similar sensitivity and specificity. As D‐2HG and L‐2HG are normal endogenous metabolites, this variable threshold for 2HG may partly reflect genetic/ethnic background and/or dietary differences. The result of our study in the US population, therefore, is important and reaffirms the diagnostic values of metabolite screening approaches to identify *IDH* mutated AML. Additional research will be helpful to further confirm and validate the diagnostic performance in larger clinical dataset.

Given *IDH* mutation producing only D‐2HG and not L‐2HG, the diagnostic performance of the enantiomeric ratio may be superior to that of D‐2HG especially at low mutant allele burden. This was suggested by two other studies [8, 9], and the findings in case 1 in our cohort in which only the D/L ratio met the diagnostic cut‐off with *IDH2* R140Q mutation at 3% VAF. This better performance may stem from the D/L ratio being technically more reliable than measuring D‐2‐HG and L‐2HG concentrations since its quantification does not require the addition of an internal standard. Future study on a large cohort is needed to evaluate the performance of the D/L ratio in monitoring therapeutic response to conventional and IDH‐targeted therapy.

While there is a general association of serum D‐2HG level and *IDH* mutation status in AML, the correlation is not perfect. A small subset of AML patients (two cases in our cohort and a small subset of AML cases in other two studies [10, 11]) had a high level of 2HG, yet no *IDH* mutation was identified by employing moderately high sensitivity molecular assays including NGS. The mechanism underlying this apparent *discordancy* remains to be investigated, including exploring other *IDH* mutation hot spots not covered by routine NGS testing, altered glutamine pathway to provide excess α‐KG, and disruption of D‐2HG dehydrogenase in AML [11, 14]. Indeed, additional D‐2HG‐producing *IDH1* mutations have been identified in non‐AML tumors, including *IDH1* R100 in glioma, *IDH1* G97 in colon cancer, and *IDH1* Y139 in glioblastoma [14].

Finally, we report the first case of increased L‐2HG without *IDH* mutation, which has not yet been described in AML. Increased L‐2HG has been reported in clear cell renal cell carcinoma due to loss or dysfunction of L‐2HG dehydrogenase (L2HGDH) to convert L‐2HG to α‐KG, which is largely associated with gene deletion on 14q, a region coding for *L2HGDH* [15]. While the relationship between increased L2HG and AML remains unclear, L‐2HG is likely an oncometabolite as L‐2HG is also an inhibitor of α‐KG‐dependent dioxygenases. The mechanism underlying such a highly increased L‐2HG in AML remains to be explored. No mutation in *L2HGDH* was detected in 10 AML patients with increased total 2HG [11]. From perspective of biomarkers, this rare finding further supports the superior diagnostic performance of D‐2HG and the D/L ratio to total 2HG as increased L‐2HG could be confounding.

In summary, our study demonstrates that elevated serum D‐2HG, total 2HG, and the D/L ratio may serve as noninvasive biomarkers of *IDH* mutation in AML. Moreover, diagnostic performance of D‐2HG and the D/L ratio may also be superior to total 2HG, particularly the D/L ratio at low mutant allele burden. Identification of elevated D‐2HG and L‐2HG in *IDH* unmutated AML should prompt investigations into novel mechanisms associated with altered metabolic activity.

## FUNDING INFORMATION

The authors received no specific funding for this work.

## CONFLICT OF INTEREST STATEMENT

The authors declare no conflict of interest.

## ETHICS STATEMENT

This study was approved by the institutional review board (IRB 122013‐023). Informed consent was not required for this study.

## PATIENT CONSENT STATEMENT

The authors have confirmed patient consent statement is not needed for this submission.

## CLINICAL TRIAL REGISTRATION

The authors have confirmed clinical trial registration is not needed for this submission.

## Data Availability

The data that support the findings of this study are available from the corresponding author upon reasonable request.
